# LEISURE ENGAGEMENT AND HAPPINESS IN LATER LIFE BASED ON A LEVEL OF FLOW EXPERIENCE

**DOI:** 10.1093/geroni/igae098.3212

**Published:** 2024-12-31

**Authors:** Jungsu Ryu

**Affiliations:** Marshall University, Huntington, West Virginia, United States

## Abstract

There has been a growing body of literature that examines various environmental predictors (e.g., time spent outdoors, and social interaction) of happiness. Furthermore, scholars have reported on the contributing role of leisure activities to happiness. However, research has rarely examined the associations between different types of leisure activity and happiness based on a different extent of flow experiences at an advanced age. Therefore, the purpose of this study was to explore what kinds of leisure activity contribute to happiness in later life based on the level of experiencing flow. A convenience sample of 188 participants was recruited from senior centers in South Korea, which consisted of 67 males and 121 females with ages ranging from 60 to 90 years (M = 74.99, SD = 5.49). The results showed that the participants in the high flow state significantly feel happier than those in the low flow state. Additionally, home-centered and social activity was the only significant contributor to happiness for the high flow state. For the low flow state, there was no significant relationship between each leisure engagement and happiness. Our findings elucidate that the importance of social engagement in happiness should be emphasized than any other activities in later life, and experiencing flow during social engagement considered meaningful also needs to be accentuated. It implies that older adults are more likely to be happy when social engagement is a meaningful and motivating activity compared to when simply participating in socializing without any motivation.

